# Factors related to childbearing intentions among women: a cross-sectional study in health centers, Saveh, Iran

**DOI:** 10.1186/s42506-020-0035-4

**Published:** 2020-02-22

**Authors:** Marzieh Araban, Mahmood Karimy, Bahram Armoon, Fereshteh Zamani-Alavijeh

**Affiliations:** 1 Department of Health Education and Promotion, Social Determinants of Health Research Center, Ahvaz Jundishapur University of Medical Sciences, Ahvaz, Iran; 2 Department of Public Health, Faculty of Health, Social Determinants of Health Research Center, Saveh University of Medical Sciences, Saveh, Iran; 3 Department of Public Health, Faculty of Health, Saveh University of Medical Sciences, Saveh, Iran; 4 Department of Public Health, Faculty of Health, Isfahan University of Medical Sciences, Isfahan, Iran

**Keywords:** Childbearing, Intention, Fertility, Psychosocial factors, Women, Iran

## Abstract

**Background:**

One of the most important demographic challenges over the recent three decades in the world has been a significant fall in the fertility rate. This study aimed to investigate factors related to childbearing intentions among a sample of Iranian women.

**Methods:**

A cross-sectional study of a sample of Iranian married women attending 8 centers in Saveh was conducted in 2015. A total of 483 married women 15–49 years old participated in this study. A questionnaire was used to collect data about demographics, attitude, subjective norms, marital satisfaction, social support, hopefulness, and behavioral intentions of childbearing.

**Results:**

Overall, 62% of women in the study intended to have children in the next 2 years. The group willing to have children had a higher score on attitude, subjective norms, hopefulness, perceived social support, and marital satisfaction compared to the group unwilling to have children. Also, the regression results revealed that the variables of age, literacy status, employment status, husband literacy, women and husband occupation status, attitude, subjective norms, hopefulness, perceived social support, and marital satisfaction were significant predicting factors for childbearing intention (*P* < 0.05).

**Conclusions and recommendations:**

The findings highlighted the importance of psychological factors such as marital satisfaction and social support in the childbearing process. Thus, health system planners should pay more attention to these determinants of fertility intention.

## Introduction

One of the most important demographic challenges over the recent three decades has been a significant fall in the fertility rate. The world’s total fertility rate has declined from 4.5 births per woman in 1970–1975 to 2.5 in 2005–2010 [1]. A wide range of social, economic, and personal factors, including reproductive behavior, higher levels of education, economic uncertainty (i.e., unemployment or employment opportunities, occupational pressure), partnership shifts (i.e., delay in delaying marriage), and economic goals or challenging housing conditions, lead young people to leave the parental home later, and this has contributed to the decrease in birth rates [1, 2]. A review (2014) indicated that parents regarded having completed an educational level, holding a job, and having a stable income and good housing are important factors for their decision-making for childbearing [3]. Another study showed that cultural factors strongly affect the parent-child relationship, which subsequently is related to reproductive behaviors [4]. However, human behaviors, including fertility behavior, are dependent on the social and cultural factors and individual and cultural differences that exist in communities and lead to different fertility behaviors [5].

Over the past three decades in Iran, the average age of marriage and having the first child has increased, and the fertility rate has dramatically declined far below the replacement level. This decline requires a comprehensive policy to manage the potential drop in population growth [1]. The World Bank has estimated Iran’s population growth rate to have dropped to 1.95% in 2010–2014 and will further drop to 1.23% in 2015–2019 and to 1.13% in 2020–2024 [2]. A study by Behboudi et al. showed that although women in Iran hold favorable attitudes toward childbearing, a wide range of socio-cultural and economic factors encourage these women to postpone their first pregnancy [6].

Behavioral theories can provide a framework to help identify beliefs that can be considered when developing an intervention [7]. Although previous studies have shown that subjective norms (SN), social support (SS), hopefulness, and marital satisfaction are associated with health behavior [2, 6, 8], to the best of our knowledge, no study has investigated the association between these theoretical factors and childbearing intention in Iran.

Because behavioral change is a complex process, a comprehensive understanding of factors related to behavioral intention is needed to help researchers and public health professionals design more effective programs. The focus of the present research was to investigate factors related to the intention of childbearing among a sample of Iranian women. The results of the current study would add to the limited body of literature addressing the issue of factors associated with childbearing intentions in Iran.

## Subjects and methods

### Participants

The present research was a cross-sectional analytic study. The study sample included married women who were residing in Saveh and Zarandieh cities in Iran in 2015–2016. The study’s inclusion criteria were being Iranian, being married for the first time, and having been married for a year or more, with no child or only one child. Women who had medical reasons for their subfertility or infertility, or were unwilling to take part in the study, were excluded.

### Procedure

After obtaining the required approval from Saveh University of Medical Sciences (UMS) and preparing a list of all healthcare centers under the jurisdiction of the faculty, research units were selected through a multi-stage sampling method. In the first stage, based on the 2013 census statistics, data were obtained regarding the population of four districts (two Nobaran central districts of Saveh and central and Kharghan district of Zarandieh) which were under the coverage of Saveh UMS. Then, from all healthcare centers of Saveh and Zarandieh, one urban and one rural center were selected via random sampling. Eventually, four urban and four rural centers were selected. In the next stage, the sample size for each center was determined based on the population covered by the center and through quota sampling. In the final stage, the required sample from each center was selected by systematic sampling method and based on the number of households in the family file. The data were available from an earlier descriptive research project.

### Sample size

Cochran’s formula was used to estimate the sample size. This formula does not estimate sample size using power analysis. By using Cochran formula, 5% margin of error, and 95% significance level, the sample size was decided to be 400 women. To increase the accuracy, 490 questionnaires were administered, of which 17 were excluded due to incompleteness, and finally, 483 questionnaires were collected (97% return rate).

### Data collection

Data were collected using a multi-section self-report questionnaire: The first part covered personal information including age, age at marriage, duration marriage, monthly household income, literacy, residential area, and employment status of women and their husbands. The other parts of the questionnaires included scales that have been widely used in the previous studies [4, 8, 9] as follows:

#### Attitudes toward childbearing and fertility [8, 10–12]

Attitudes of women toward childbearing and fertility were assessed using 15 items on a Likert type scale ranging from 1 (completely agree) to 5 (completely disagree), (e.g.: In my opinion/belief, life without having any children is dull and spiritless). The total range of scores for this section was from 15 to 75 with the higher values indicating better attitudes respectively. The reliability of the attitude scale yielded a satisfactory level of Cronbach’s alpha (0.81).

#### Subjective norms (SN) [12–14]

SN, which refer to the perceived pressure of important others to perform or not to perform a behavior, were assessed using 6 items, (e.g.: My husband thinks that one child is enough). A scale from completely correct (1) to completely incorrect (5) was used to assess these SN. The total possible range of scores in this section was 6 to 30, with the higher values indicating better SN. Internal consistency of the scale was measured by Cronbach’s alpha, and the results revealed a good level of reliability (0.77).

#### Behavioral intention of childbearing [8, 12–14]

The behavioral intention was defined as a woman’s perceived likelihood that she would engage in childbearing. It was evaluated through four 5-point items, including “I will definitely do this (5), I most probably will do this (4), I may do this (3), I possibly will not do this (2), and I will not do this at all (1)” (e.g., “At any time during the next 2 years do you intend to get pregnant?”). The scores of this part ranged from 4 to 20, and its reliability was tested with Cronbach’s alpha (0.85).

#### The ENRICH Marital Satisfaction Scale [15]

The ENRICH Marital Satisfaction Scale included 35 items ranked based on a 5-point Likert scale, namely, the style responses of “absolutely agree,” “agree,” “neither agree nor disagree,” “disagree,” and “totally disagree”; these items scored from 1 to 5. In this questionnaire, the evaluated satisfaction aspects are as follows: personal issues, leisure activities, sexual relationships, family and friend’s religious orientation, and parenting. The scores of this part ranged from 35 to 175. In Iran, the scale content validity and reliability were calculated and confirmed [16]. In our study, good reliability was confirmed by a Cronbach’s alpha level of 0.81.

#### The Snyder Hopes Scale [17]

The Snyder Hopes Scale included 8 items ranked based on an 8-point scale from completely wrong (1) to completely right (8), (e.g., “I can find many ways to achieve the things that are important for me”). The minimum and maximum scores were 8 and 64, respectively. A higher score in the scale showed a higher level of hope. The questionnaire has two sub-scales called hope agency and hope pathways. In Iran, psychometric properties of the scale has been satisfactory [18]. In our study, good reliability was confirmed with a Cronbach’s alpha of 0.78.

#### Social support (SS) [19]

The multidimensional scale of perceived SS, which is a 12-item questionnaire developed by Zimet et al., 2013 measures the perceived SS from family, friends and significant others. This instrument provides response options ranging from 0 to 6 (very strongly disagree to very strongly agree). The scores of this part ranged from 0 to 72. A higher score reflects more support. The reliability and validity of the Farsi version of the perceived SS have been evaluated [20]. In the present study, Cronbach’s alpha for the scale was 0.89.

### Validity

To ensure the selected items were of the best quality, quantitative and qualitative content validity ratio (CVR) and content validity index (CVI) were used. This process included asking 10 experts in the areas of health education, social medicine, public health, midwifery, and obstetrics to divide the items into three categories of “necessary,” “beneficial but not necessary,” and “not necessary.” Based on the Lawche’s table, the items with CVR > 0.62 were considered as significant and therefore; they all remained in the questionnaire (*P* < 5%). For the purpose of CVI estimation, experts were asked to rate scales for relevancy and clarity based on a 4-point Likert type scale. Items were regarded as clear and relevant if they obtained values equal to or greater than 0.79. Consequently, all items remained within the questionnaire. To check the qualitative content validity, 10 experienced university professors were asked to assess the quality of included items and consider the grammatical features, wording, item placement, and grades assigned to each item.

### Data analyses

The Kolmogorov-Smirnov test was used to determine the normal distribution of the obtained data. Data were analyzed by SPSS 18 (SPSS, Chicago, IL, USA, acquired by IBM) using Chi-square, independent sample *t* tests, Pearson correlation, and regression tests. *P* < 0.05 was considered as statistically significant. To assess factors associated with childbearing intent, multiple logistic regression analyses were conducted to generate odds ratios (OR) and 95% confidence intervals (CI) for the associations of interest. Only the independent variables that showed significant associations with childbearing (*P* ≤ 0.05) in bivariate analyses were included in the multiple logistic regression model.

To determine the dependent variable in logistic regression models, data regarding fertility intention status within 2 years were collected using one question: “During the next 2 years do you have an intention to have a child?” with yes/no responses. Participants who indicated that they did not have any intention to have a child in the next 2 years were coded 0, and the others as code 1.

## Results

A total of 490 women were included in this research but 483 women returned completed questionnaires. The age of the respondents (*n* = 483) ranged from 17 to 43 years, with a mean of 24.7 ± 2.7 years. Women aged 17 to 25 years had the highest risk ratio and those at older ages (35 to 41 years) were less likely to intend childbearing. None of the participants had a child, and 86% were housewives. Regarding their education, the majority of the women were high school (66.4%) (Table 1).

**Table 1:** Demographic variables of women attending healthcare centers of Saveh and Zarandieh, Iran, 2015–2016

Variables	Childbearing intention		No intention for childbearing		*P*^a^
	*N* = 300 (62%)	%	*N* = 183 (38%)	%	
Age					
17–25	47	15.7	83	45.4	0.001
26–34	102	34	68	37.1	
35–43	151	50.3	32	17.5	
Employment					
Employed	39	13	29	15.8	0.05
Housewife	261	87	154	84.2	
Husband’s employment					
No	4	1.3	11	6	0.005
Yes	296	98.7	172	94	
Education					
University	51	17	45	24.6	0.06
High school	206	68.6	115	62.8	
Elementary and illiterate	43	14.4	23	12.6	
Husband’s education					
University	91	30.3	78	42.6	0.01
High school	157	52.3	84	45.9	
Elementary and illiterate	52	17.4	21	11.5	

^a^Chi-square

Sixty-two percent of the participants said that they wanted to have children in the next 2 years. The majority of them (69%) preferred to have only two children. Twenty-two percent of the participants wanted three or more children. The majority of participants (87%) believed that the age of 25–29 years was an appropriate age for the first childbearing. Only a small percentage (18%) wanted to have their last baby between the ages of 35 and 39 years (Table 2).

**Table 2:** Women preferences for the number of, and the time for having their children

Variable	Total		Childbearing intention		No intention for childbearing		*P*^a^
	*N* = 483	%	*N* = 300 (62%)	%	*N* = 183	%	
The desired number of children							
1	60	12.4	27	9	33	18	0.009
2	333	69	211	70.3	122	66.7	
≥ 3	90	18.6	62	20.7	28	15.3	
Appropriate age for the first childbearing							
18–24	11	2.3	7	2.3	4	2.2	0.52
25–29	421	87.1	256	85.3	165	90.2	
30–34	51	10.6	37	12.4	14	7.6	
Age for the last pregnancy							
25–29	301	62.3	189	63	112	61.2	0.94
30–34	95	19.7	59	19.7	36	19.7	
35–39	87	18	52	17.3	35	19.1	

^a^Chi-square

**Fig. 1:**
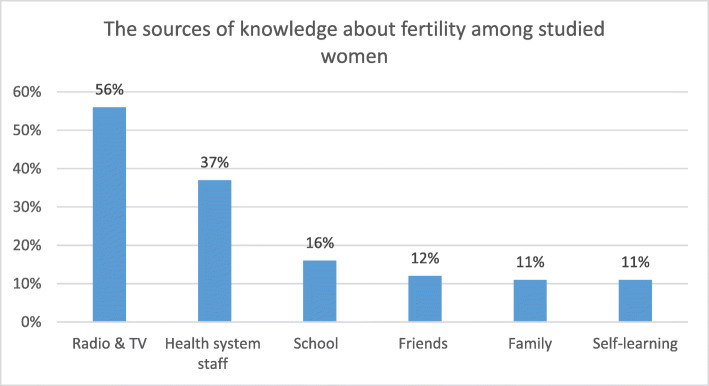
The sources of knowledge about fertility among studied women

The majority of the participants (58%) stated that they were “somewhat educated” about fertility topics, and they had gained most of their knowledge from radio and TV (56%), health system staff (37%), school (16%), friends (12%), family (11%), and self-learning through reading resources such as journals and books (11%)(Fig. 1).

Independent *t* tests showed a significant difference between the mean scores of attitude, SN, hopefulness, perceived SS, and marital satisfaction of women who had the intention to childbearing and those who had no intention for childbearing (*P* < 0.05) (Table 3).

**Table 3:** The mean scores and standard deviation of psychological factors influencing the childbearing intentions of women

Variables (score range)	Desire to childbearing		No intention for childbearing		*P*^a^
	Mean	SD	Mean	SD	
Attitude (15–75)	55.1	10.4	42.1	18.3	0.001
Subjective norms (6–30)	23.9	6.3	19.2	7.1	0.05
Social support (0–72)	49.4	8.3	38.9	9.1	0.001
Hopefulness (8–64)	43.3	8.5	37.6	9.2	0.001
Marital satisfaction (35–175)	38.2	7.6	31.4	8.7	0.001

^a^Independent *t* tests

Pearson correlation test results indicated that age was negatively correlated with fertility intention; therefore, with an increase in age, fertility intent decreased (*r* = − 0.39 and *P* = 0.02). Attitude (*r* = 0.45), SN (*r* = 0.41), marital satisfaction (*r* = 0.38), marital duration (*r* = 0.36), hopefulness (*r* = 0.42), and perceived SS (*r* = 0.49) were positively correlated with fertility intention (*P* < 0.05) (Table 4).

**Table 4:** Correlations between the childbearing intention and psychological factors

Variable	Childbearing intention	Attitude	Subjective norms	Social support	Hopefulness	Marital satisfaction	Marital duration
Childbearing intention	1						
Attitude	0.45*	1					
Subjective norms	0.41*	0.28*	1				
Social support	0.49*	0.37*	0.31*	1			
Hopefulness	0.42*	0.08	0.19	0.18	1		
Marital satisfaction	0.38*	0.33*	0.21	0.09	0.27*	1	
Marital duration	0.36*	0.11	0.06	0.24*	0.16	0.19*	1

*Correlation is significant at the 0.05 level

Factors such as age, literacy status, residential area, occupation, husband occupation status, husband literacy, monthly income, urban and rural roots, attitude, SN, hopefulness, perceived SS, and marital satisfaction were positively associated with childbearing intention in the univariate analyses. The multiple unconditional logistic regression analysis revealed that a number of demographic variables (age, literacy status, employment status, husband literacy, husband’s occupational status), attitude, SN, hopefulness, and perceived SS were significant factors related to pregnancy and childbearing intention. Marital satisfaction, perceived social support, and hopefulness were factors with the highest odds for childbearing intent (OR = 3.51, 3.47, 2.14, respectively) (Table 5).

**Table 5:** Results of the multiple logistic regression analysis of psychological and demographic factors

Variables	*B*	Std. error	Wald	Sig.	Exp(*B*)	95% confidence Interval for Exp(*B*)	
						Lower	Upper
Attitude	0.42	0.15	3.60	0.01	1.52	1.08	2.26
Subjective norms	0.65	0.47	4.71	0.001	1.95	1.45	2.68
Social support	0.74	0.21	4.46	0.001	2.14	1.69	2.72
Hopefulness	1.24	0.69	5.23	0.001	3.47	2.10	5.24
Marital satisfaction	1.25	0.70	6.42	0.001	3.51	2.65	5.89
Sociodemographic variables							
Age							
35–43	Ref.				Ref.		
26–34	0.48	0.30	6.35	0.001	1.62	1.22	2.09
17–25	0.61	0.26	5.81	0.001	1.85	1.35	2.37
Employment							
Employed	Ref.				Ref.		
Housewife	0.37	0.11	8.89	0.001	1.42	1.12	1.77
Husband’s employment							
No	Ref.				Ref.		
Yes	0.83	0.30	7.31	0.001	2.28	1.35	2.56
Education							
University	Ref.				Ref.		
High school	0.22	0.14	2.43	0.03	1.26	1.02	1.58
Elementary and illiterate	0.34	0.23	2.30	0.01	1.41	1.07	1.92
Husband’s education							
University	Ref.				Ref.		
High school	0.30	0.21	2.28	0.001	1.35	1.18	1.84
Elementary & illiterate	0.46	0.25	3.34	0.001	1.59	1.20	2.01

## Discussion

Based on our results, 62% of the participants reported they were likely to have children in the next 2 years. This finding is in line with the results of Keshavarz et al. (Iran) in which the fertility preference on average was 59% [21]. In a study conducted by Hoseini and Bagi (Iran) [22], the percentage was 41%. However, in a study by Lampic et al. in Sweden [23], this rate was 96.5%. Similarly, the results of Kerzer and White (Italy) showed that about 28% of participants tended to want to have children [24]. This inconsistency in the results may be due to the differences in sampling and the definition of “childbearing desire” used in the different studies. For example, the Swedish study was among university students of both sexes who had chosen a longer degree program, and thus do not tend to postpone childbirth until they have earned their degrees, about half of them were unmarried. The question asked was not including any time orientation, just “Do you plan to have children?” (Yes/No) (of course the majority will respond by “Yes”), while all women in our sample were married and we asked them about their intentions in the next 2 years (62% of study sample intended to have children within the next 2 years).

The present study indicated that marital satisfaction was one of the factors positively associated with childbearing intention. This result supported by previous studies [25, 26]. Previous research has indicated that SS, as a social determinant of health, plays an important role in promoting healthy conditions in people’s lives. Similar to our study, findings have been reported by other studies in Germany [13], Iran [14], and in four European countries [27]. According to SS theories, relationships are not necessarily sources of SS unless the people perceive them as available and suitable sources of support for their needs [28].

Researchers have shown that individuals with higher levels of hope have better performance in objects related to health maintenance and problems. Similarly, our findings revealed that hopefulness was significantly associated with childbearing intentions. Other studies [4, 29] showed that hopefulness influenced deciding and timing of the childbearing and more hopeful women were willing to have a child sooner, which were consistent with the findings of the our study.

In line with previous researches on different populations [12, 30], the results of our study indicated that more positive attitudes toward having a child were associated with more favorable intentions to have a child. Most of the literature on the TPB-based framework, emphasize the importance of attitude as a basic influential construct in the prediction of behavior [7, 8, 26]. In this study, SN of the women were significantly associated with their childbearing intention. Similarly, previous studies showed that SN may play a key role in low fertility contexts [8, 12, 13, 30]. According to the TPB, SN in the childbearing intentions is linked to the increased importance of individual self-sufficiency.

Our findings showed that age was significantly and negatively associated with childbearing intention. This finding is consistent with existing evidence showing that age has an effect on childbearing [4, 29, 30]. For instance, a study by Kodzi et al. showed that with each 1-year increase in age at the first childbearing, fertility dropped 3% in women [31].

Our results showed that fertility intention decreased with more education and employment of women. This is in line with the common evidence available on the role of education and employment status in childbearing [4, 32, 33]. Previous studies have also shown that men and women regard having completed an educational level, holding a good job, and having a good income as important factors affecting their decision to become parents [1, 4, 34]. For instance, Virtala et al. found that younger women’s educational, occupational, and career goals were of more importance, and they may delay childbearing to achieve these other priorities [35]. Similarly, our findings showed that men’s and women’s occupational status were significantly associated with their childbearing intentions. Women’s increased educational level and occupation postponed their age at first marriage and improved their socio-economic status, and all of these factors were associated with decreased fertility.

### Strengths and limitations

The results of the current study would add to the limited body of literature addressing the issue of key factors associated with childbearing intentions. A few limitations should be taken into account while interpreting the findings of this study. First, the results of this study cannot be generalized to women attending private clinics and centers because they were not represented in the study sample. In addition, the data were cross-sectional; thus, temporal and potential causal relationships cannot be inferred. Future studies are recommended to determine predicting factors in decision-making for childbearing intentions in couples (men and women).

## Conclusions and recommendations

Attitude, social norms, hopefulness, perceived social support, and marital satisfaction were all significantly associated with childbearing intention. Moreover, demographic factors, including age, occupational status, and literacy were significant factors related to childbearing intention. These findings highlight the importance of some personal and psychosocial factors in the childbearing process. Thus, health system planners should pay more attention to these determinants during development of programs.

## Data Availability

Upon request, we can offer onsite access to external researchers to the data analyzed at Saveh University of Medical Sciences, Saveh, Iran.

## Abbreviations

CVI: Content validity index
CVR: Content validity ratio
SN: Subjective norms
SS: Social support
